# Diagnostic role of aortic valve calcium scoring in various etiologies of aortic stenosis

**DOI:** 10.1038/s41598-023-34118-7

**Published:** 2023-05-17

**Authors:** Wisarut Wanchaitanawong, Rungsrit Kanjanavanit, Tanop Srisuwan, Wanwarang Wongcharoen, Arintaya Phrommintikul

**Affiliations:** 1 Division of Cardiology, Department of Internal Medicine, Faculty of Medicine, Chiang Mai University, Chiang Mai, 50200 Thailand; 2 Department of Radiology, Faculty of Medicine, Chiang Mai University, Chiang Mai, Thailand

**Keywords:** Cardiology, Diagnosis

## Abstract

Most of the studies about aortic valve calcium (AVC) score in aortic stenosis (AS) were based on degenerative or bicuspid AS but not rheumatic AS. We aimed to study the diagnostic accuracy of AVC score to determine severe AS in various etiologies. Adult patients diagnosed with mild to severe AS were enrolled. AVC score were identified from multi-detector computed tomography (MDCT) scan. The AVC score was highest in bicuspid AS (3211.9 (IQR (1100.0–4562.4) AU) compared to degenerative AS (1803.7 (IQR (1073.6–2550.6) AU)), and rheumatic AS (875.6 (IQR 453.3–1594.0) AU), *p* < 0.001. For the ROC curve to identify severe AS, the AVC score performed well in degenerative and bicuspid AS with the area under the ROC curve (AuROC) 0.834 (95% CI, 0.730, 0.938) in degenerative group; and 0.820 (95% CI, 0.687, 0.953) in bicuspid AS. Whereas AVC score had non-significant diagnostic accuracy with AuROC 0.667 (95% CI, 0.357, 0.976) for male and 0.60(95% CI, 0.243, 0.957) for female in rheumatic AS. The cut-off AVC score values to identify severe AS were AVCS > 2028.9AU (male) and > 1082.5AU (female) for degenerative AS, and > 2431.8AU (male) and > 1293.5AU (female) for bicuspid AS. In conclusions, AVC score is the accurate test for assessing severity in patients with degenerative and bicuspid AS but performs poorly in rheumatic AS group.

## Introduction

Aortic valve stenosis (AS) is the most common valvular heart disease with an increasing prevalence in aging population^1^. Although degenerative calcific aortic stenosis is the most common cause in the western population, rheumatic heart disease remains common in developing countries^2^. Aortic valve calcification is a common pathological finding in various causes of aortic stenosis including degenerative aortic stenosis, bicuspid aortic valve as well as rheumatic aortic stenosis^3^. Previous studies showed the different pattern of aortic valve calcification in different causes of aortic stenosis. In degenerative aortic valve, the calcification begins at the base of the cusps and progresses towards the edges, while relative sparing the commissures. In contrast, the commissural fusion and fibrosis, with stiffen and retraction of aortic valve cusps are presented in rheumatic aortic valve disease. In bicuspid valve disease, calcification takes place mainly on the cusps^3^. The degree of calcification increased by time and significantly contribute to obstructive physiology of the valve^2^.

The association between computed topographic aortic valve calcium (CT-AVC) score and hemodynamic measurements of stenosis severity on echocardiogram has been demonstrated in several studies^4–8^. Koos et al. found aortic valve calcification scores were correlated significantly with peak-to-peak and mean transvalvular gradients measured by cardiac catheterization^5^. Nevertheless, women developed severe aortic stenosis with apparently less calcium^5–7^. The gender-specific CT-AVC threshold have been proposed to identify severe AS (1274 and 2065 Agatston unit for women and men respectively)^9^. In addition, the CT-AVC can be used to predict disease progression and adverse clinical events^8^. It is useful for clinical decision in patients with paradoxical low flow low gradient aortic stenosis, and low flow low gradient AS with no demonstrable flow reserve^10^. However, there are some limitations in clinical use of CT-AVC. Most of studies on aortic valve calcium score in AS were derived from patients with degenerative or bicuspid aortic stenosis without population of rheumatic heart disease. Therefore, this study aimed to demonstrate diagnostic accuracy of CT-AVC score in determining severe AS in various etiologies. The pattern to aortic valve calcification in various causes of aortic stenosis were also to be studied.

## Methods

This is a cross sectional study, conducted in Maharaj Nakorn Chiang Mai Hospital during January 2015–January 2021. The study was approved by an institutional review board along with the name of the IRB, and that the participants gave written informed consent. We recruited 134 patients in our study. The patients aged ≥ 18 years with mild, moderate and severe AS diagnosed with echocardiogram were included. The patients were excluded from the study if they had endocarditis, suspected paradoxical low flow—low gradient AS, incomplete /non-diagnostic echocardiographic image quality, and pregnant women. All patients had comprehensive echocardiographic studies. The multi-detector computed tomography (MDCT) scans were performed within a period of 3 months after echocardiogram.

The echocardiogram were analyzed by 2 independent cardiologists. If the results were discordance, the third cardiologist would make a joint decision for identifying etiology and severity. The echocardiographic views and image quality grades were defined based on current IAC Standards and guidelines published by the American Society of Echocardiography (ASE) and the American College of Cardiology (ACC)^11^. The definition of AS severity was defined according to 2020 ACC/AHA Guideline for the Management of Patients with Valvular Heart Disease which mild, moderate and severe stenosis defined as Aortic Vmax 2.0–2.9, 3.0–3.9 and $\ge 4$ m/s or mean transaortic valve pressure gradient < 20, 20–39 and $\ge$ 40 mm Hg for mild, moderate and severe AS respectively^12^.

For the MDCT scan, all patients were scanned using a third-generation dual source CT scanner (SOMATOM Force, Siemens, Germany). The non-contrast prospective ECG-gating transaxial scan of the heart, between level of tracheal carina and diaphragm, were performed by using ultra-high-pitch spiral acquisition, tube voltage of 120 kV with adaptive tube current. The images were reconstructed by using a standard filtered-back projection algorithm with 3-mm slice thickness and 2.5-mm slice increment. The acquisition were placed from the bottom of the valve to the level of the sino-tubular junction (STJ). AVCS and coronary calcium score (CCS) were determined 2-dimensionally by using the calcium score data sets on the Syngovia workstation and defined by Agatston units (AU). The AVCS area of interest comprised the valve cusp and aortic annulus. The CT scan results were finalized by experienced cardiovascular radiologist including aortic and coronary calcium score value and pattern of calcification in various etiologies of aortic stenosis and other findings.

### Statistical analysis

All statistical analyses were performed using SPSS for window version 23. Continuous data were reported as a mean with standard deviation ± SD for parametric data and as a median with interquartile range (IQR) for non-parametric data. Between-group comparisons were performed using the Kruskal–Wallis test for non-parametric data or the one-way ANOVA test for parametric data. If the aforementioned tests showed a significant difference, a post-hoc pairwise comparison with a Bonferroni correction was done to find a significantly different pair. Categorical data were expressed as number (percentage of the total). Between-group comparisons were performed using Pearson's bivariate test and the χ^2^ test for categorical covariates, and Student T-test or one-way analysis of variance for continuous covariates. Receiver operator characteristic (ROC) curves were derived to assess diagnostic accuracy of CT-AVC and to identify the optimum thresholds for severe AS diagnosis. All statistical tests were 2-sided, and p values of < 0.05 were considered statistically significant.

### Ethical approval

Aortic Valve Calcium Scoring in various etiologies of Aortic Stenosis among Thai patients was approved by the ethics committee of the Faculty of Medicine, Chiang Mai University, approval number 134/2563 for protocol MED-2563-07035, version 2.0. The investigations were carried out in accordance with the Declaration of Helsinki, including written informed consent of all participants.

## Results

### Baseline clinical characteristics

A total of 134 patients (66 males) were included in this study. Clinical baseline characteristics are shown in Table 1. There were 17 (12.7%), 37 (27.6%) and 80 (59.7%) with mild, moderate and severe AS respectively. Among patients with mild AS, the CT-AVC was studied in addition to the other indications for CT such as aortic disease or coronary artery disease. There were 64 patients (47.4%) with degenerative AS, 43 patients (31.9%) with bicuspid AS, and 27 patients (20.1%) with rheumatic AS. Patients with the degenerative AS were significantly older than patients with bicuspid AS and rheumatic AS (77.9 ± 7.5 vs. 64.1 ± 13.0 vs. 60.3 ± 13.1 years; *p* < 0.001 respectively). There was no significant difference regarding gender, body weight, diabetes, hypertension, smoking status among groups. Patients with degenerative AS had significant higher prevalence of dyslipidemia than other groups (65.6% vs. 46.5% vs.25.0% in degenerative AS vs. bicuspid AS vs. rheumatic group respectively, *p* = 0.001). The prevalent atrial fibrillation was significantly highest in the rheumatic AS (46.5% vs.12.5% vs. 9.4% in rheumatic AS vs. degenerative AS and bicuspid AS respectively, *p* =  < 0.001).

**Table 1:** Patients’ characteristics.

	Degenerative AS (N = 64	Bicuspid AS (N = 43)	Rheumatic AS (N = 27)	*p* value for multiple groups comparison
Age (yr.)	77.9 ± 7.5	64.1 ± 13.0*	60.3 ± 13.6*	< 0.001
Male (N, %)	33 (51.6)	20 (46.5)	13 (48.1)	0.945
Body weight (kg)	61.6 ± 14.4	60.7 + 14.9	58.3 ± 9.9	0.66
Hypertension (N, %)	49 (76.6)	31 (72.1)	15 (55.6)	0.135
Diabetes mellitus (N, %)	16 (25.0)	6 (14.0)	6 (22.2)	0.332
Dyslipidemia (N, %)	42 (65.6)	20 (46.5)	7 (25.9)	0.001
History of Smoking (N, %)	15 (26.3)	9 (21.0)	6 (22.2)	0.09
Atrial fibrillation (N, %)	8 (12.5)	4 (9.4)	13 (48.1)	< 0.001

*AS* aortic stenosis.

Data presented as mean ± SD or N (%) *p < 0.05 compared to degenerative AS in post hoc analysis.

### Echocardiographic data

The average trans-aortic valve maximum velocity (V_max_) and mean pressure gradient (MPG) were significantly higher in the bicuspid AS (V_max_ = 4.28 ± 1.16 m/s, MPG = 51.8 ± 26.3 mmHg), than other groups (V_max_ = 3.79 ± 0.85 m/s, MPG = 37.6 ± 18.0 mmHg for degenerative AS and Vmax = 3.73 ± 0.72 m/s, MPG = 33.9 ± 13.2 mmHg for rheumatic AS), *p* < 0.001. There was no significant difference of mean aortic valve area, mean stroke volume and left ventricular ejection fraction (LV EF) among groups. However, patients in rheumatic AS had lower proportion of low LVEF (LVEF < 50%). The mean ascending aortic diameter was significantly largest in bicuspid AS group (3.65 ± 0.74 cm) compared to degenerative AS and rheumatic AS (3.15 + 0.47 and 3.14 + 0.46 cm, respectively), *p* = 0.003 (Table 2).

**Table 2:** Echocardiographic data.

Parameters	Degenerative AS (N = 64)	Bicuspid AS (N = 43)	Rheumatic AS (N = 27)	*p* value for multiple groups comparison
Aortic V_max_ (m/s)	3.79 ± 0.85	4..28 ± 1.16*	3.73 ± 0.72#	0.018
Mean pressure gradient (mmHg)	37.6 ± 18.0	51.8 ± 26.3*	33.9 ± 13.2#	< 0.001
Stroke volume (ml)	66.3 ± 18.1	66.3 ± 18.1	69.1 ± 17.4	0.747
Aortic valve area (cm^2^)	0.85 ± 0.32	0.75 ± 0.39	0.94 ± 0.30	0.135
LV EF (%)	63.1 ± 16.4	59.1 ± 16.9	66.45 ± 8.8	0.053
LVEF < 50%, N (%)	13 (20.6)	14 (31.8)	1 (3.7)	0.018
Severity of AS				0.042
Mild, N (%)	9 (14.1)	4 (9.3)	4 (14.8)	
Moderate, N (%)	16 (25.0)	8 (18.6)	13 (48.1)	
Severe, N (%)	39 (60.9)	31 (72.1)	10 (37.0)	

*AS* aortic stenosis, *LVEF* left ventricular ejection fraction.

Data presented as mean ± SD or N (%).

*p < 0.05 compared to degenerative AS in post hoc analysis.

#*p* < 0.05 compared to bicuspid AS in post hoc analysis.

### Computed tomographic data of calcium score and patterns of calcium distribution

Computed tomographic data of calcium score and patterns of calcium distribution are presented in Table 3. The median aortic valve calcium score was significantly highest in bicuspid AS (3211.9 (IQR (1100.0–4562.4)) AU) compared to degenerative AS (1803.7 ((1073.6–2550.6)) AU), and rheumatic AS (875.6 ((453.3–1594.0)) AU), *p* < 0.001). The pattern of calcification was different among groups. There was significantly higher prevalence of aortic annular and aortic valve leaflet calcification in degenerative and bicuspid AS than in rheumatic AS (*p* = 0.005). The rheumatic AS had significantly higher prevalence of predominately aortic commissural calcification (59.2%), mitral annular calcification (77.8%), mitral valve leaflet calcification (59.2%) and LA wall calcification (30.4%) than other groups (*p* < 0.001).

**Table 3:** Computed tomographic data of calcium score and pattern of calcium distribution.

Parameters	Degenerative AS (N = 64)	Bicuspid AS (N = 43)	Rheumatic AS (N = 27)	*p* value for multiple groups comparison
Aortic valve calcium score (Agatston unit), median (IQR)	1803.7 (1073.6–2550.6)	3211.9 (1100.0–4562.4))*	875.6 (453.3–1594.0)#	< 0.001
Aortic annulus calcification, N (%)	51 (79.6)	35 (86.0)	13 (48.1)	0.003
Aortic valve leaflet calcification, N (%)	64 (100.0)	38 (88.3)	20 (74.1)	0.001
Aortic valve commissure calcification, N (%)	62 (98.4)	39 (90.9)	7 (25.9)	< 0.001
Aortic valve commissural predominance calcification, N (%)	0	3 (6.9)	16 (59.2)	< 0.001
Mitral annulus calcification, N (%)	46 (71.8)	12 (27.9)	21 (77.8)	< 0.001
Mitral valve leaflet calcification, N (%)	9 (14.1)	2 (4.6)	16 (59.2)	< 0.001
Left atrial wall calcification, N (%)	2 (3.1)	1 (2.3)	7 (30.4)	< 0.001

Data presented as median (interquartile range) or N (%).

*p < 0.05 compared to degenerative AS in post hoc analysis.

#for *p* < 0.05 compared to bicuspid AS in post hoc analysis.

### Aortic valve calcium score for identifying severe aortic stenosis

**Figure 1:**
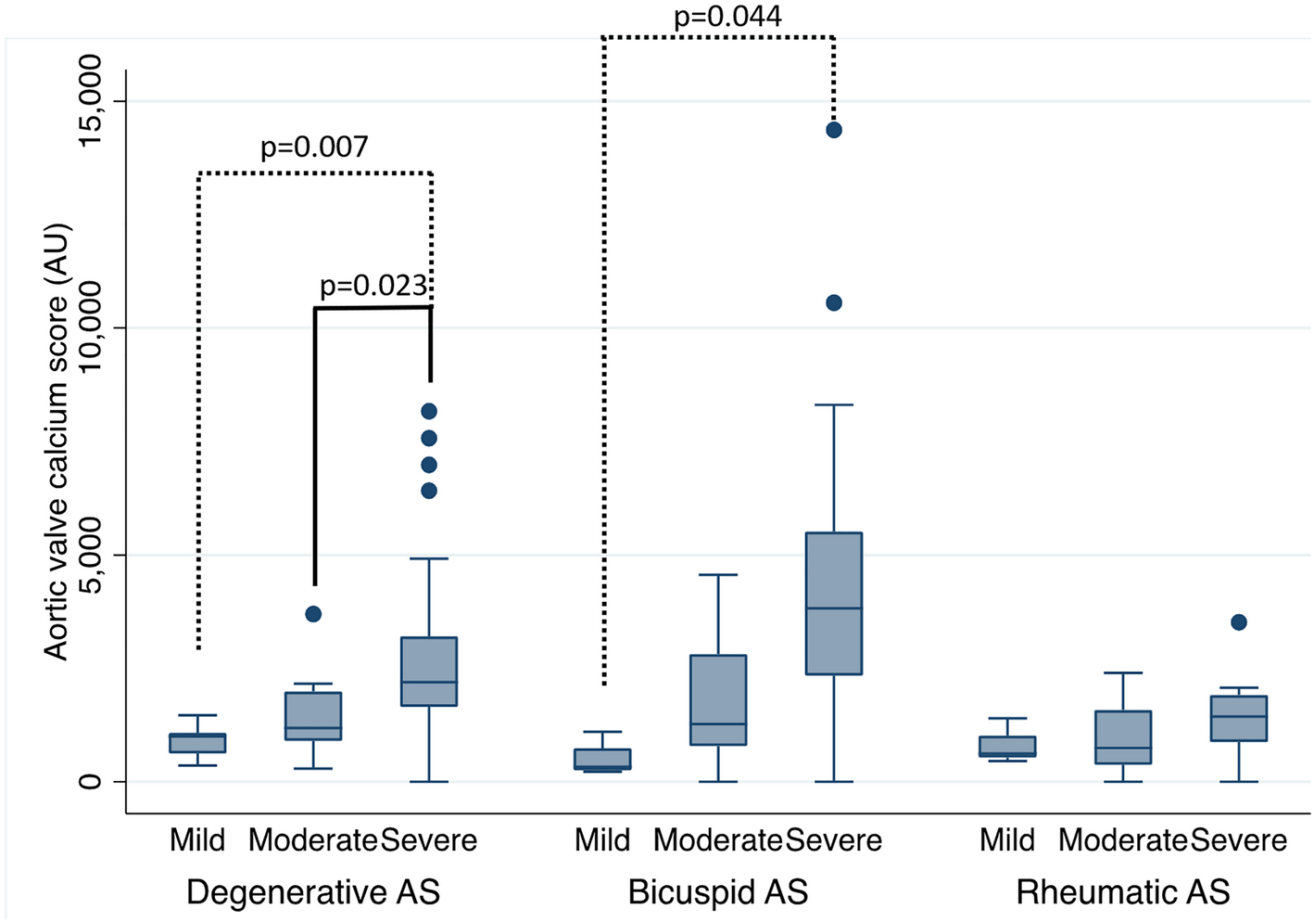
CT-Aortic valve calcium score by severity in the various etiologies. (The box indicates the 25th to 75th percentiles, and asterisk*/small circle o is outlier). AS = aortic stenosis.

The CT-AVC score by severity in the various etiologies are shown in Fig. 1. Among patients with degenerative AS, the medians of CT-AVC score were significantly highest in severe AS (997.1 (IQR 577.2–1195.2) AU for mild AS, 1184.6 (IQR 876.1–2049.5) AU for moderate AS, and 2206.0 (IQR 1649.6–3244.7) AU for severe AS, *p* < 0.001). In bicuspid group, the medians of CT-AVC score were significantly highest in severe AS (328.6 (IQR 235.5–917.0) AU for mild AS, 1273.2 (IQR 670.5–3130.1) AU for moderate AS, and 3839.0 (IQR 2380.8–5513.9) AU for severe AS), *p* = 0.009). Interestingly, in rheumatic group, the medians of CT-AVC score were not significantly different between severe and non-severe AS. (619.5 (IQR 429.4–1205.4) AU for mild AS, 745.0 (IQR 216.4–1596.5) AU for moderate AS, and 1433.0 (IQR 822.2–1951.5) AU for severe AS), *p* = 0.245).

Among 64 patients with severe aortic stenosis from various etiologies. The median CT-AVC score was significantly highest in bicuspid group (3839.0 (IQR 2380.8–5513.9) AU) compare to degenerative group (2206.0 (IQR 1649.6–3244.2) AU) and rheumatic group (960.1 (IQR 822.2–1951.5) AU, *p* < 0.001).

### Diagnostic accuracy and cutoff point for assessing severity in aortic stenosis by using aortic valve calcium score

**Figure 2:**
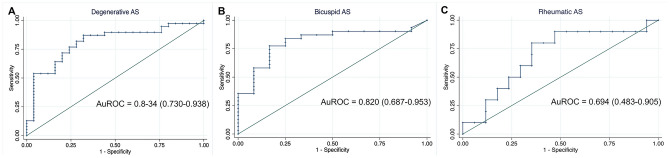
Receiver operating characteristic curve to identify severe aortic stenosis in various etiologies. (**A**) Degenerative aortic stenosis. (**B**) Bicuspid aortic stenosis. (**C**) Rheumatic aortic stenosis.

**Figure 3:**
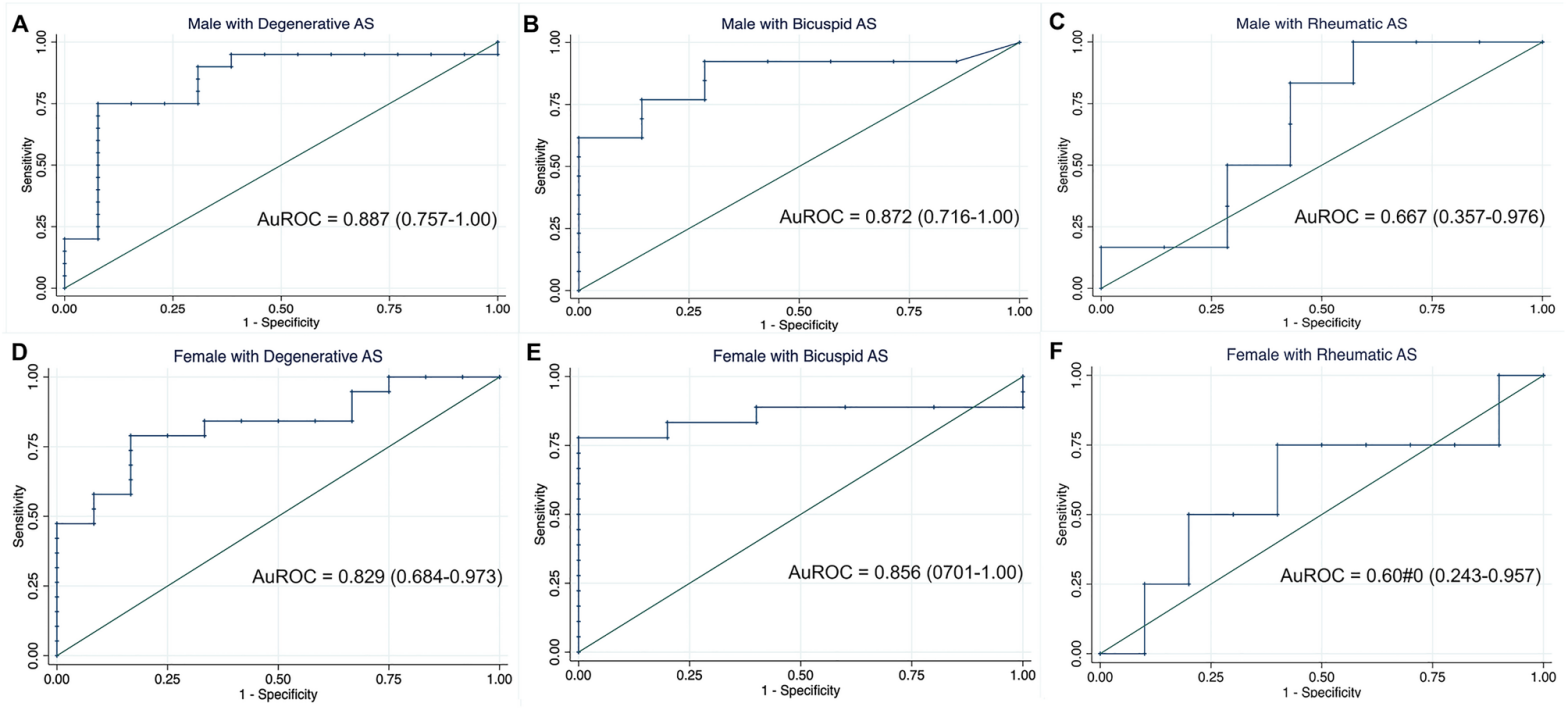
Receiver operating characteristic curve to identify severe aortic stenosis in various etiologies discriminate by gender. (**A**) Degenerative aortic stenosis in men. (**B**) Bicuspid aortic stenosis in men. (**C**) Rheumatic aortic stenosis in men. (**D**) Degenerative aortic stenosis in women. (**E**) Bicuspid aortic stenosis in women. (**F**) Bicuspid aortic stenosis in women.

The ROC curves were constructed to identify severe aortic stenosis for each etiology (Fig. 2) and gender (Fig. 3). The AVC score had significant diagnostic accuracy in degenerative and bicuspid group with the area under the ROC curve as following: 0.834 (95% CI, 0.730, 0.938) for all, 0.887 (95% CI, 0.757, 1.00) for male, .and 0.829 (95% CI, 0.684, 0.973) for female in degenerative group; and 0.82 (95% CI, 0.687, 0.953) for all, 0.872 (95% CI, 0.716, 1.00) for male and 0.856 (95% CI, 0.701, 1.00) for female in bicuspid group. Whereas the rheumatic group had the lowest area under the ROC (0.694 (95% CI, 0.483, 0.905)) for all, 0.667 (95% CI, 0.357, 0.976) for male and 0.60 (95% CI, 0.243, 0.957) for female.

The best cutoff values to identify severe AS in various etiologies (Table 4) were AVCS > 2146.4 AU in male and > 1316.6 AU in female for degenerative group, AVCS > 3532 AU in male and > 1483.9 AU in female for bicuspid group. Due to poor test performance, optimal cutoff value is not justified in rheumatic group.

**Table 4:** Cut-point and accuracy of aortic valve calcium score for identifying severe aortic stenosis (by gender).

Gender	AUC		Threshold (AU)	Sensitivity (%)	Specificity (%)	PPV (%)	NPV (%)
Women	Degenerative AUC = 0.829 (95% CI, 0.684, 0.973)	Specific	1641.2	57.9	91.7		
		Best	1082.5	84.2	66.7	80.0	72.7
		Sensitive	631.35	100	25.0		
	Bicuspid AUC = 0.856 (95% CI, 0.701, 1.00)	Specific	1293.5	77.8	100		
		Best	1145.0	83.3	80	78.9	66.7
		Sensitive	807.1	88.9	60		
Men	Degenerative AUC = 0.887 (95% CI, 0.757, 1.00)	Specific	2168.4	78.9	92.3		
		Best	2028.9	89.5	70.0	80.9	81.8
		Sensitive	1651.1	100	61.5		
	Bicuspid AUC = 0.872 (95% CI, 0.716, 1.00)	Specific	4997.3	64.3	100		
		Best	2431.8	92.9	71.4	86.7	83.3
		Sensitive	2431.8	92.9	71.4		
All patients	Degenerative AUC = 0.834 (95% CI, 0.730, 0.938)	Specific	2168.4	55.3	96		
		Best	1556.1	81.2	72	80.0	72.7
		Sensitive	1112.5	92.1	56.0		
	Bicuspid AUC = 0.820 (95% CI, 0.687, 0.953)	Specific	3532.7	69.4	91.7		
		Best	1483.9	84.4	75.0	90.0	64.3
		Sensitive	1034.8	90.6	50.0		

## Discussion

This cross-sectional study main findings showed that in patients with degenerative and bicuspid aortic stenosis, CT-AVC score is the accurate test for identifying severe aortic stenosis but poor performance in rheumatic aortic stenosis group. For pattern of calcification from MDCT, we found that higher prevalence of aortic annular and aortic valve leaflet calcification in degenerative and bicuspid groups. However, the rheumatic group had significantly higher prevalence of predominately aortic commissural calcification, mitral annular calcification, mitral valve leaflet calcification, and left atrial wall calcification. The prevalence of atherosclerosis and median coronary calcium score was significant higher in degenerative group than other groups.

Although aortic valve calcification play a major role in obstructive physiology, it has been shown in this study that patients with rheumatic AS could have severe AS without heavily calcified valves (median AVCS in rheumatic AS group (960.1 (IQR 822.2–1951.5 AU) vs. bicuspid group (3839.0 (IQR 2380.8–5513.9) AU) and degenerative group (2206.0 (IQR 1649.6–3244.2) AUAU). Similar to the previous finding that calcification constitutes a major feature of aortic stenosis in degenerative and bicuspid group, though to a lesser extent in rheumatic group^13^. In addition to valvular calcification, commissural fusion may also contribute to the severity of stenosis in rheumatic heart disease.

The poor diagnostic performance in rheumatic aortic stenosis could also be contributed to the relatively younger population compared to bicuspid and degenerative aortic stenosis. This finding is supported by the previous study which demonstrated the absent of aortic valve calcification by fluoroscopic study in rheumatic aortic stenosis in patients under age 30 years^14^. The effects of age in calcification was also seen in our bicuspid population. There were 2 young patients (19 and 25 years old) with severe bicuspid aortic stenosis who had very low aortic valve calcium score (CT-AVC score 0 and 107 AU). These patients had extensive commissural fusion with doming valve without discernible calcification seen by echocardiogram.

Comparing CT-AVC score between degenerative AS and bicuspid AS, our finding is consistent with previous study which showed higher mean CT-AVC score of bicuspid group than tricuspid aortic stenosis^15^. The hypothesis that could explain this finding is that the mechanical forces are less efficiently distributed in bicuspid aortic valve. The exposure to higher tensile stress may provoke early and rapid progression of aortic valve calcification^16^. Roberts et al.^17^ reported in that bicuspid aortic valve patients had more severely calcified and remodeled valves than tricuspid aortic valve patients.

For the pattern of calcium distribution, there was significantly higher prevalence of aortic annular and aortic valve leaflet calcification in degenerative and bicuspid groups than in rheumatic group. These findings could be explained that in degenerative aortic stenosis, calcified nodules are initially observed at the base (annulus) of the cusps and gradually extends towards the orifice^18^. In bicuspid aortic valve, when leaflets undergoes degeneration, calcification tends to predominate along the fusion line and the base of the conjoined leaflets^19^. In contrast, rheumatic AS group had significantly higher prevalence of predominately aortic commissural calcification, mitral annular calcification, mitral valve leaflet calcification, and LA wall calcification than other groups consistent with prior knowledges^20,21^. In our study, the medians of aortic valve calcium score of male are higher than female in all etiologies. Several studies have also shown that female manifest lower AVC than male after taking the effect of smaller body size, heart and aortic annulus size in to account^9,22–25^. Intrinsic contrasts between valves from male and female have been exhibited at cellular and genetic levels^26,27^. This was postulated that fibrotic process is more extensive in women than men. Therefore, valvular mobility could be limited without extensive calcification^28^.

Clavel et al.^9^ proposed the threshold for identifying severe aortic stenosis (women 1274 AU and men 2065 AU) and Pawade et al.^24^ eventually proposed nearly identical threshold (women 1377 AU and men 2062 AU). In our study, the best threshold for identifying severe degenerative AS performed similarly well as previous studies (women 1082 AU and men 2029 AU) and demonstrate excellent area under the ROC curve (0.829 for women and 0.887for men) but reproducibility and generalizability of these CT-AVC thresholds is only for degenerative group. From Clavel et al. data^9^, CT-AVC score is recommended for aortic valve severity evaluation^10^. Our data showed the best cutoff value for severe bicuspid aortic stenosis (women 1145 AU, Men 2431 AU) with outstanding area under the ROC curve (0.858 for women and 0.872 for men). The cutoff level was comparable with the previous studies. Choi et al. Demonstrated the cut-off value of 1423 AU in women and 2573 AU in men with AUC of 0.8^23^. Similarly, Boulif et al. demonstrated mean value of AVCS was 3534 ± 1777 AU^15^, and Ren et al. (3497.71 ± 2470.17 AU)^29^.

## Strength and study limitations

This is the first study that demonstrate the cutoff value of aortic valve calcium score identifying severe aortic stenosis in various etiologies which included rheumatic AS group. Nevertheless, this study has several limitations which should be acknowledged. First, it was a single center cross-sectional study. Second, we carefully discriminated the etiologies of aortic stenosis in multiple views by echocardiography and confirmed by one or two experienced cardiologists. Nevertheless, we cannot exclude that a few patients could have been misclassified, especially in heavy calcified patients. Third, the sample size in our study was small, we need confirmation of a larger group of patients especially in rheumatic group. Fourth, we excluded the patient with paradoxical low flow low gradient severe AS from our study. So caution need to be exercised when the findings are to be generalized in this group of patients. Further studies in this group of patients are warranted.

## Clinical applications

Our study has confirmed the value of aortic valve calcium score for identifying severe aortic stenosis in nonwestern population especially in degenerative and bicuspid AS. The best cut off values of degenerative AS are 1082 AU for women and 2029 AU for men, consistent with current practice guidelines. For bicuspid AS, the best cut off value are 1483 AU for women and 2431 AU for men. Younger patients (especially in women) with severe bicuspid and rheumatic aortic stenosis had very low level of aortic valve calcium score. Therefore, due to poor test performance, we do not recommend using AVCS in classification of AS severity in this subset of patients.

## Conclusion

In patients with degenerative and older bicuspid aortic stenosis, CT-AVC score is the accurate test for assessing severity but performs poorly in rheumatic aortic stenosis. Due to the limited sample size, further study with larger population is warranted.

## Data Availability

The datasets used and/or analysed during the current study available from the corresponding author on reasonable request.
